# An Audit of Malignant Solid Tumors in Infants and Neonates

**Published:** 2012-01-01

**Authors:** Sushmita N Bhatnagar

**Affiliations:** Department of Paediatric Surgery, B.J. Wadia Hospital for children, Parel, Mumbai 400 012, India

**Keywords:** Infant, Solid tumors, Malignancy, Audit

## Abstract

Background: To audit the demographics, outcome and factors affecting long-term survival in infants and neonates with solid tumors.

Material and Methods: Retrospective case series was performed for 13 years. Demographics, surgical notes, treatment protocols and outcome details were reviewed.

Results: Of total 372 tumors over 13 years, there were 59 infants (15.86%) of which 8 were neonates, with M:F 1.2:1, and mean age of presentation was 5.18months. Fifty three of the infants had tumors which were > 5 cm in size. Thirty two (54%) had a rapid progression of the lesion during investigations. Tumors markers and pre-operative biopsy were diagnostic in 61.5% and 30% respectively. Neuroblastoma was the commonest tumor (22%), followed by hepatoblastoma (20.3%), malignant germ cell tumor (20.3%), soft tissue sarcomas (11.9%), and others (8.5%). Staging distribution for 39 (66%) infants showed Stage 1-n=9, Stage 2-n=15, Stage 3-n=7, Stage 4-n= 5 and Stage IVs-n=3. Nineteen (32.2%) babies received chemotherapy. Almost half (50.8%) of the children underwent surgical removal of the tumor; with gross total resection in 76.6%. The overall mortality was 35.6%. About 30.5% are alive, well and tumor free on 2-12 years follow-up.

Conclusion: A much higher incidence (15.8%) of infantile tumors in our region as compared to literature (2%) is alarming. Treatment failures from deaths or non-compliance amounted to be 69.5%. These are the two major issues which need to be addressed in the future management of infantile tumors. Reduction in deaths due to chemotherapy toxicity, rapid surgical intervention and R0 resection and risk stratification needs to be incorporated, to improve long-term tumor free survival in infants.

## INTRODUCTION

Solid tumors are uncommon in neonatal and infantile periods as compared to their counterpart in older children. This group of tumors behaves differently in terms of etiopathogenesis, response to therapy and behavior patterns as well as long term outcomes; hence it is imperative to study these tumors as separate entity [1].

Two-thirds of the neonatal tumors are diagnosed in the first week of life, comprising 2% of childhood malignancies. Infantile solid tumors account for 10% of malig¬nancies seen in children. The highest reported incidence is in Japan with no sex predilection. Neuroblastoma (NB), Wilm’s tumor (WT), teratoma and soft tissue sarcomas (STS) rank amongst the most common tumors in neonates and infants. Other tumors include hepatoblastoma, Central Nervous System neoplasms and retinoblastoma. Information regarding timing of presentation and diagnosis as well as outcome especially amongst neonates is limited owing to rarity of cases [1,2]. The goal of the article is to audit the demographics and outcome in infants with solid tumors treated in a tertiary care pediatric hospital in India.

## MATERIALS AND METHODS

A retrospective case series was performed in the Department of Pediatric Surgery by retrieving data from files of all infants who were treated in our institute for solid tumors between January 1996 and December 2009. Age and sex distribution of the patients were collected. The mode of diagnosis, whether antenatal or post natal, was ascertained in all patients presenting with solid tumors in the neonatal age. Data obtained from the records included the type and location of tumors, histological diagnosis (preoperative or post operative), presence or absence of metastatic disease, and treatment protocols. The distribution of the solid tumors in the infantile age group and their outcomes were reviewed.

## RESULTS

In 59 study cases, 33 were boys (56%) and 26 girls (44%). The age at presentation ranged from day 1 of life to 12 month with a mean age of 5.18 months (Table 1).

**Figure:**
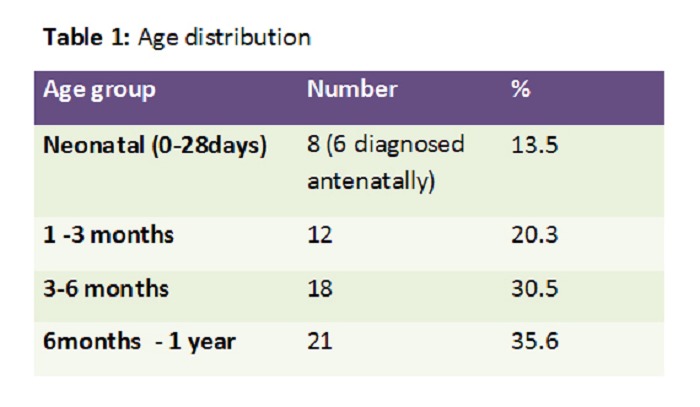
Table 1: Age distribution

Clinically; almost all of the infants except for 2 had a palpable lump on presentation. While the infants were in the process of being investigated, almost half [n=32(54%)] of them showed a rapid increase in size of the lesion. All infants were investigated with initial ultrasonography and combined with CT scan or MRI. Specific diagnostic workup included tumors markers in 39 (66.1%) and pre-operative biopsy in 18 (30%). Size of the tumor at presentation was classified into two groups: less than 5 cm which was found in 4 (7%) of patients, and >5 cm in 51(93%). Tumor markers such as Alpha-fetoprotein was done in 24 infants and was found to be raised in 18 with 10 hepatoblastomas, 4 sacrococcygeal teratomas, 1 retroperitoneal teratomas, 1 ovarian tumor, and 2 hemangioendotheliomas. Urinary VMA done in 13 suspected neuroblastomas was found to be raised in 8, thus being diagnostic in 61.5% of these.

Table 2 shows the tumor distribution amongst the study group, neuroblastoma being the most common tumor followed by malignant germ cell tumor and hepatoblastoma (Table 2). Location of malignant germ cell tumors comprised of Sacrococcygeal Teratoma (8/12) (Fig. 1), retroperitoneal (2/12) and one each involved the testis and ovary. Of the 12 cases of hepatoblastomas, two had neonatal presentations. The renal tumors seen in our series were 10(16.9%), of which two were congenital mesoblastic nephromas; followed by 7 infants with soft tissue sarcoma (11.9%), 4 rhabdomyosarcoma, one each myxoid and undifferentiated type of non-rhabdomyosarcomatous STS, and one infantile fibrosarcoma (Fig. 2). The other rare tumors seen were 4 haemangioendotheliomas and a single case of pancreatoblastoma.

**Figure:**
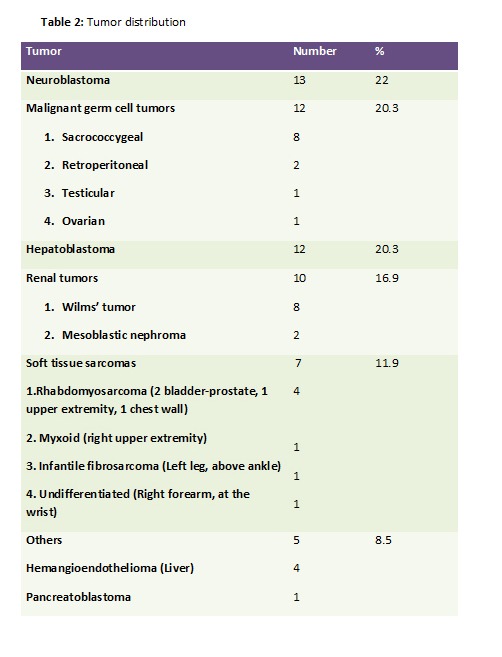
Table 2: Tumor distribution

**Figure:**
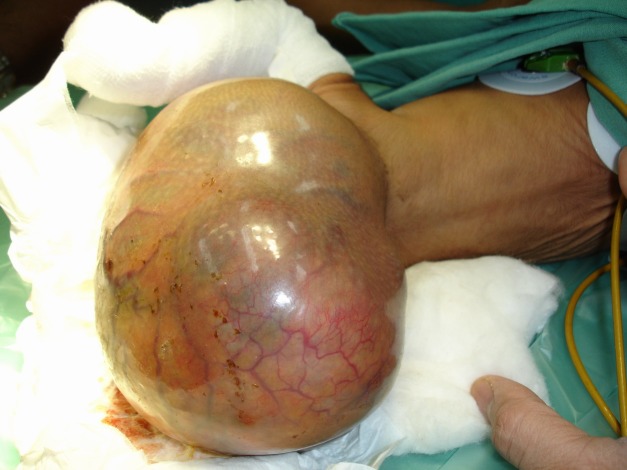
Figure 1: Sacrococcygeal teratoma

**Figure:**
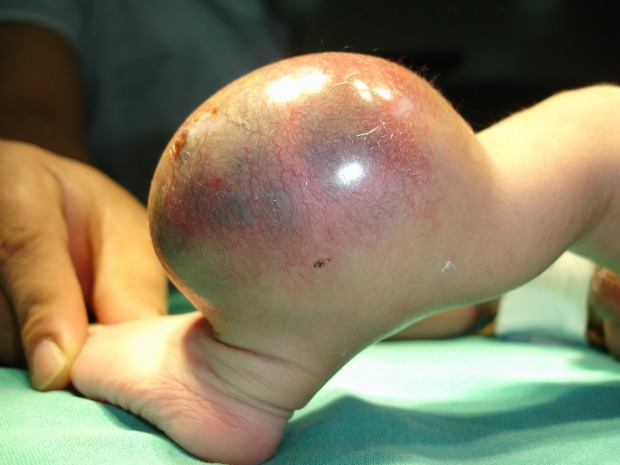
Figure 2: Fibrosarcoma

Complete staging could be performed in 39 (66%) patients which showed a majority being in the Stage 2 category; the detailed distribution being Stage 1=9(23.07%), Stage 2=15(38.46%), Stage 3=7 (17.94%), Stage 4= 5 (12.82%) and Stage IVs=3(7.69%).

Management of all these infants with solid tumors was done jointly in the Departments of Pediatric Surgery and Pediatric Oncology at our institute. Nineteen (32%) infants received chemotherapy. Thirty of the 59 (50.8%) infants underwent surgical interventions, of which gross total resection (GTR) was done in 23 (76.6%), incomplete excision (macroscopic residual) in 3(10%), while, in 4 infants only open biopsy was done. In the group of children who had GTR, 5 had positive surgical margins on histopathology, and all 5 were thence treated with salvage chemotherapy and radiotherapy in one patient with NB. Table 3 describes the type of resection and its outcome in the 30 infants who underwent surgical intervention (Table 3).

**Figure:**
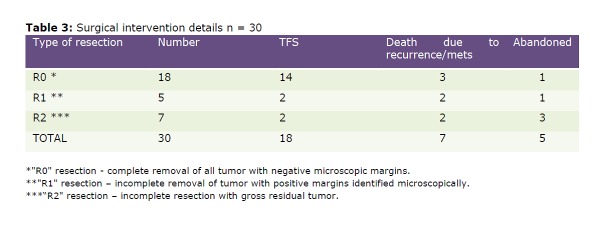
Table 3: Surgical intervention details n=30

Several major chemotherapy related complications were identified such as febrile neutropenia n=23 (episodes), hepatitis/liver failure n=7, congestive cardiac failure n=1 and nephrotic syndrome n=2. Analysis of the over­all outcome led to the findings as tabulated (Table 4). Thus, there were 20/59 (33.9%) who abandoned treatment, 21/59 (35.6%) succumbed to treatment, leaving only 18/59 (30.5%) surviving and well with follow-up ranging from 2-12 yrs.

**Figure:**
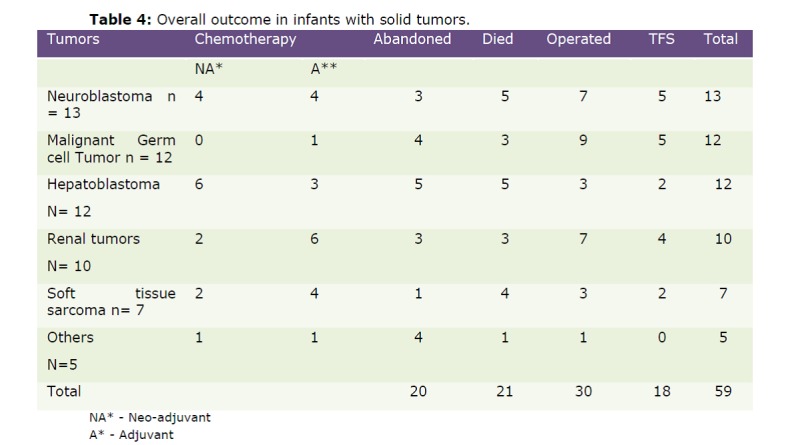
Table 4: Overall outcome in infants with solid tumors

## DISCUSSION

Solid tumors in the infants are a distinct entity, especially those in the neonates. Even though the neonatal tumors are more commonly benign, there are a sizeable number which are malignant or behave like malignancy and these pose a therapeutic challenge. Hence, the malignant solid tumors in infants should be categorized differently as similar pathologies behave and respond in a disparate manner. As compared to their counterparts which occur in older children, these have different behavior patterns and hence a thorough histological and cytogenetic evaluation is essential for better outcome. The guidelines for management for these tumors essentially remains the same, except for reduction in the chemotherapeutic doses as the tissues and organs are immature and thus are extremely sensitive to the toxicities of anticancer treatment [1-3].

On compilation of our experiences in the infantile tumors, a significant demographic difference was noticed in the incidence of neonatal malignancies. Worldwide incidence of neonatal tumors is about 2%, the highest incidence being in Japan and our series showed 13.5% incidence. This study being a retrospective analysis, the details of causation of increased incidence of infantile tumors could not be assessed [1,4,5].

As regards the type of the tumor, most of the series have a preponderance of NB and malignant GCT as the most common solid tumors in infancy, though there are regional variations [11,12]. CNS tumors and retinoblastomas are not included in our study as our Department of Pediatric Surgery does not manage these lesions [2,6-12]. Surgery has been the mainstay of treatment in infants in our study, especially because of the nature of the tumors. With a large number of infants being denied treatment by their parents, the number of babies who actually underwent R0 resection was just 30.5%. As noted in this study and by other authors, the behavior of solid tumors in the infantile period is not always similar to their counterparts in older age groups [9]. In our series, the number of recurrences and metastases was definitely higher especially in the malignant GCT and renal tumor groups, thus reducing the long term tumor free survival in these groups which otherwise would have had a better prognosis. Though there were no major surgery related complications causing mortality, there were some children who died following surgery, due to chemotherapy related complications.

Due to abandonment of treatment and chemotherapy related mortality the number of children in each group who are alive and tumor free is miniscule. About 5/13 (38.5%) of NB are surviving. On literature review, infantile NB seem to be having a good prognosis, wherein all the infants are surviving and well as reported by Kaneko in 2005 [13,14]. Similarly, the malignant GCT group showed a tumor free survival of 5/12 (41.6%), renal tumors 4/10 (40%), STS 2/7 (28.6%), hepatoblastomas 2/12 (16.6%) and the rare tumors 0/5 (0%) long term overall survival. In spite of having a large series with 59 infants in the study group, it is unfortunate to see less than 50% infants surviving in any of the tumor categories, partly contributed by complications of chemotherapy in spite of dose reduction as per age; the complications being febrile neutropenic episodes, nephropathy, cardiopathy and liver injury.

Overall outcome in the literature showed 75-80% survival for all types of infantile tumors while our study had overall TFS of a meager 30.5%.

Multiple factors are responsible for this poor outcome which includes gender bias, poor socioeconomic strata, poor nutritional status, low birth weight coupled with increased risk of febrile neutropenic episodes and hospital acquired infections, patients coming from distant villages that neither afford nor complete the treatment course.

## CONCLUSION

Treatment failures from deaths or non-compliance to therapy amounted to a staggering 69.5%. These are the two major issues which need to be addressed in the future management of infantile tumors in our institution. Most importantly, we need to reduce the chemotherapy related mortality by improving the standard of care being offered to neonates and infants on chemotherapy.

## Footnotes

**Source of Support:** Nil

**Conflict of Interest:** None declared

